# How an Aggressive Weedy Invader Displaces Native Trees

**DOI:** 10.1371/journal.pbio.0040173

**Published:** 2006-04-25

**Authors:** Liza Gross

Humans have never been known to tread lightly on the earth, but as our global reach has expanded so have our impacts on other species. Vanishing habitat caused by human activity is the number one threat to biodiversity, but the dispersal of alien invasive species—again, caused by humans—is not far behind. Over 4,500 non-native plant and animal species have established residence in the United States since European settlement, according to a 1993 report by the US Office of Technology and Assessment. Many alien species cause little disturbance, while others radically transfigure their new habitat by displacing less competitive native species and disrupting fragile ecological relationships that evolved over millions of years.

Of a growing list of invasive plants in North America, garlic mustard *(Alliaria petiolata)* has been on the Nature Conservancy's Red Alert list since 2000. Originally found in Europe, it was planted in the late 1860s by European settlers for its medicinal and culinary properties. The weed has since spread from New York to Canada and 30 US states in the East and Midwest, with recent sightings as far west as Oregon. Many mechanisms have been proposed to explain the success of alien plant invasions, mostly related to the absence of natural predators or parasites or the disruption of long-established interactions among native organisms. Few studies, however, have directly tested these possibilities. In a new study, Kristina A. Stinson, John N. Klironomos, and colleagues do just that by investigating garlic mustard's effects on native hardwood North American trees. The weed gains a competitive advantage, they discovered, by releasing chemicals that harm a fungus the trees depend on for growth and survival.

Many forest trees and other vascular plants form mutually beneficial relationships with arbuscular mycorrhizal fungi (AMF). The fungus has long filaments that penetrate the roots of plants (forming branched structures called arbuscules) and snake through the soil in an intricate interwoven network of mycelium, which effectively extends the plant's root system. AMF depend on the plant for energy, and the plant depends on the fungus for nutrients. Many non-native plants, including garlic mustard, do not depend on native AMF and often take root in landscapes altered by development or logging, where AMF networks are disturbed. When these non-mycotrophic invasives propagate, they may diminish AMF densities even further.

Biologists are especially concerned about what might happen if a non-mycorrhizal invasive plant turns up in a mature, intact forest with an established mycelial network—which is just what garlic mustard has started to do. In the North American forests it has recently invaded, the plant inhibits the growth of understory plants, including the seedlings of canopy trees. Stinson et al. suspected the invader might somehow be thwarting the symbiotic relationship between fungus and tree.

To test this possibility, they collected soil from five forests in Ontario dominated by four species of native hardwoods. Soil was taken from infested and uncontaminated areas from each location. First, the researchers tested seedlings' ability to form mycorrhizal relationships in soil with a history of garlic mustard invasion. Three species—sugar maple, red maple, and white ash—had significantly less AMF root colonization and slower growth when grown in the infested soil. Seedlings grown in sterilized soil taken from invaded and pest-free locations showed similar reductions, suggesting that diminished microbial activity led to suppressed growth.

A second set of experiments supported this conclusion by showing that native trees grown in soils conditioned with garlic mustard (weeds were grown in soil, then removed) had lower AMF colonization and impaired growth than when grown in soil conditioned by native plants. Since adding extracts of garlic mustard impaired AMF colonization and seedling growth as effectively as the whole plants did, the researchers concluded that garlic mustard uses phytochemical poisons to disrupt native plants' mycorrhizal associations and stunt their growth.

Stinson et al. go on to show that garlic mustard's impacts vary with a native plant's AMF dependency. Plants with fewer roots to take up nutrients—like the hardwood seedlings studied here—will be most affected by garlic mustard invasions. This suggests that garlic mustard is invading the understory of mature forests because it's poisoning the lifeblood of its woody competitors. If true, the appearance of this noxious weed in an intact forest promises to have devastating impacts. First the plant will stifle the regeneration of the dominant canopy trees, and then it will pave the way for weedy plants that don't like the beneficial fungi.

Which phytochemicals are to blame and how they interact with other beneficial soil microbes is a question for future study. Determining if and how plants in garlic mustard's native European habitat peacefully coexist may suggest ways to help North American natives fend off its fungicidal attacks. With evidence that the plant can displace native species within ten years of establishing a presence, prudence suggests taking steps to eradicate the weed before all the answers are in.

## 

**Figure:**
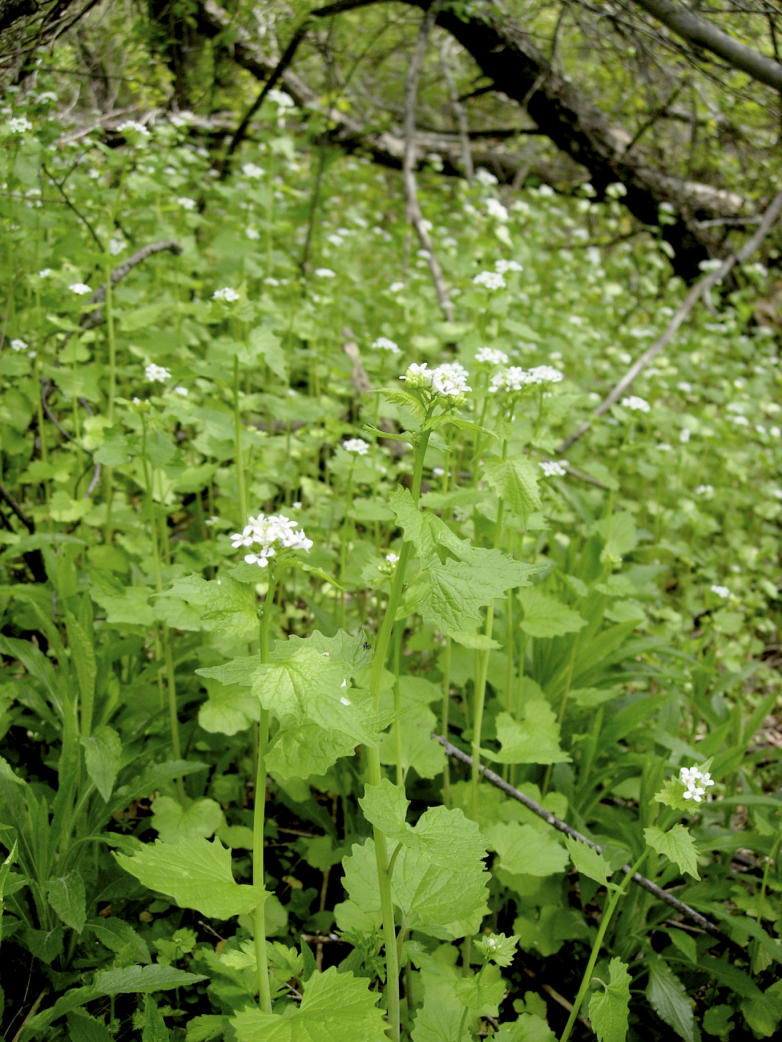
Severe invasion by garlic mustard in a maple-dominated forest understory in New England. (Photo: Benjamin Wolfe)

